# Fever of Unknown Origin in a 17-Year-Old Girl

**DOI:** 10.7759/cureus.10607

**Published:** 2020-09-23

**Authors:** Yanki K Okuducu, Adaku Nwosu, Ahmed Awad, Ratna B Basak

**Affiliations:** 1 Pediatrics, Brookdale University Hospital Medical Center, New York, USA

**Keywords:** fever of unknown origin, pediatric fevers of unknown origin, fever, pyrexia, pyrexia of unknown origin

## Abstract

Fever of unknown origin (FUO) is defined as fever (>101°F) that lasts more than three weeks and for which a cause is not found within seven days of hospital evaluation. FUO has a broad list of differentials - infection, inflammatory diseases, and malignancy. A detailed history and meticulous clinical examination with thorough and stepwise investigations lead to a diagnosis in only two-thirds of cases.

In this article, we present a 17-year-old adolescent girl, with no significant past medical history, who presented with FUO during the COVID pandemic. A high index of suspicion and extensive investigations revealed the final diagnosis.

## Introduction

With hundreds of potential causes, FUO is challenging for physicians. In recent times, COVID-19 has been recognized as a cause of persistent fever [1].

During the peak of the COVID-19 pandemic, a 17-year-old adolescent girl presented with high fever for three weeks and was suspected of having multisystem inflammatory syndrome in children (MIS-C) after initial laboratory tests. She tested negative for polymerase chain reaction (PCR) COVID-19 multiple times. Although it is well-known that PCR COVID-19 can be negative in children with MIS-C [2-3], a search for an alternate cause led to the final diagnosis.

## Case presentation

A 17-year-old adolescent girl with no significant past medical history presented to the emergency room with complaints of a high fever (102-103°F), sore throat, headache, watery diarrhea, dizziness, and palpitations for the past three weeks. She denied cough, shortness of breath, joint pains, skin rashes, night sweats, history of recent travel, exposure to sick contacts, or illicit drug use.

Her mother reported anorexia with an unintentional weight loss of 8 pounds during this period. The patient had an emergency room visit for left knee pain three months prior, which had subsequently improved with analgesics. All immunizations were up to date except for the annual influenza vaccine.

The physical examination showed a thin, ill-looking adolescent with a fever (102.7°F), tachycardia (130/min), and hypotension (82/42 mmHg), with normal respiratory rate and oxygen saturation.

There were no rashes, joint swelling, tonsillar exudates, organomegaly, heart murmur, or added sounds upon lung examination. The initial laboratory findings are summarized in Table 1.

**Table 1:** Laboratory data during admission WBC: white blood cell; RBC: red blood cell; HBG: hemoglobin; BUN: blood urea nitrogen; ALT: alanine transaminase; AST: aspartate aminotransferase; LDH: lactate dehydrogenase; CRP: C-reactive protein; TSH: thyroid-stimulating hormone

Data	WBC	RBC	HBG	Hematocrit	Platelet Count	Sedimentation Rate	Glucose	BUN	Creatinine	Sodium	Potassium	Chloride	CO_2_	Calcium	Uric Acid	Protein, Total	Albumin	Bilirubin, Total	ALT	AST	Alkaline Phosphatase	LDH	CRP	TSH	Free T4
Measurement	3.60(L)	3.45(L)	10.8(L)	33.2(L)	142(L)	127(H)	121 (H)	7	0.63	130(L)	3.3(L)	100	25	7.2(L)	2.6	6.1(L)	2.1(L)	0.3	75(H)	144(H)	66	3,578(H)	7.40(H)	3.95	0.58(L)
Ref Range	4.50 - 10.20 10x3/Ul	4.10 - 5.10 10x6/uL	11.4 - 15.5 g/dL	37.0 - 49.0 %	180 - 401 10x3/uL	0-20 mm	70 - 99 mg/dL	7.0 - 17.0 mg/Dl	0.52 - 1.04 mg/dL	133 - 145 mEq/	3.5 - 5.1 mEq/L	98 - 107 mEq/L	22 - 30 mEq/L	8.4 - 10.5 mg/dL	2.5 - 6.2 mg/dL	6.3 - 8.2 g/dL	3.5 - 5.0 g/dL	0.2 - 1.3 mg/dL	9 - 52 U/L	14 - 36 U/L	38.0 - 126.0 U/L	313 - 618 IU/L	0.50 - 1.00 mg/dL	0.465 - 4.680 mIU/L	0.78 - 2.19 ng/dL

**Figure 1:**
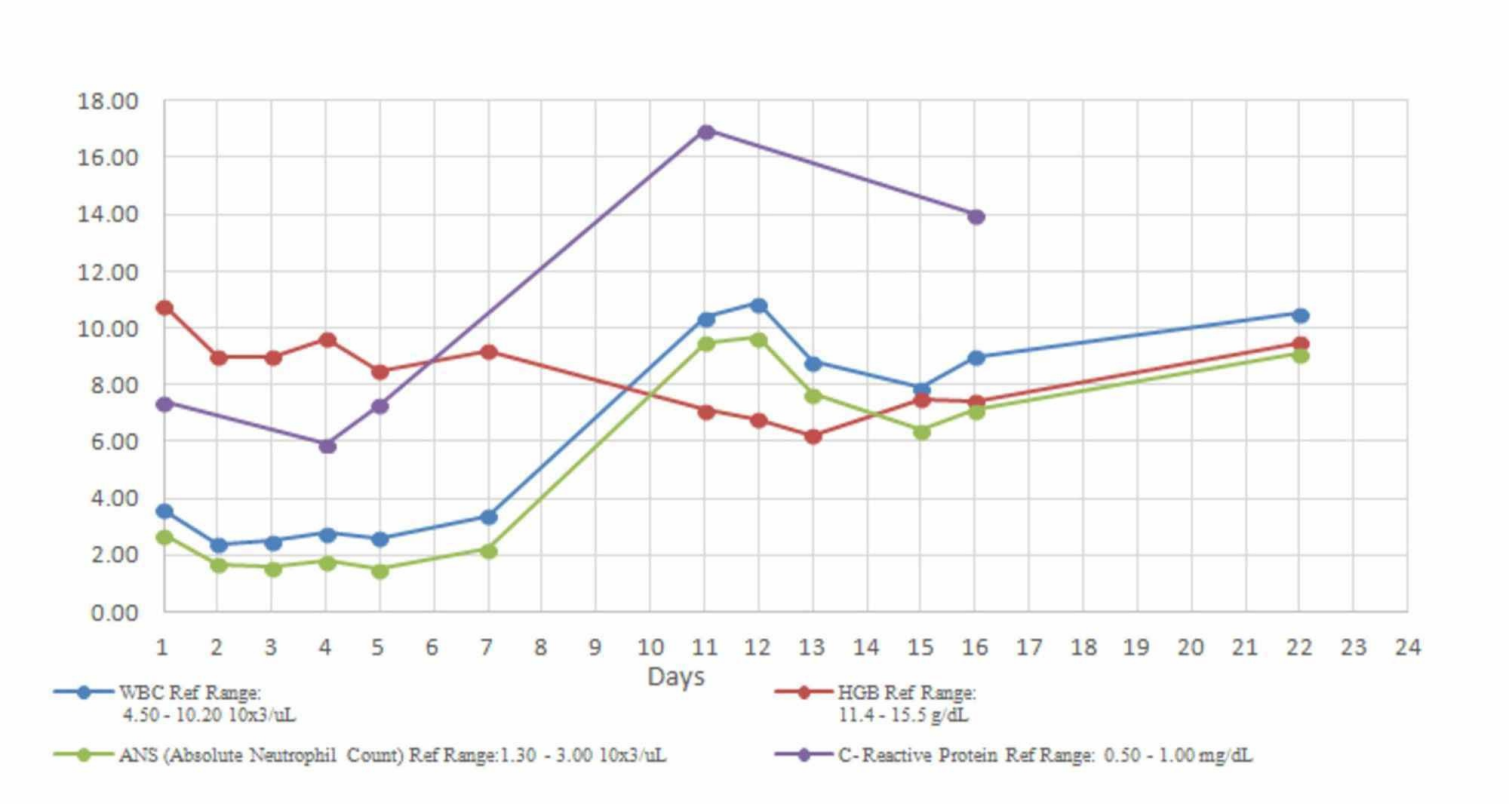
Trends of WBC count, ANS, HBG, and CRP with time WBC: white blood cell; ANS: absolute neutrophil count; HBG: hemoglobin; CRP: C-reactive protein

During the next two weeks, our patient continued to have a high fever of up to 103-104°F. All workup for common and atypical infections, which included Cytomegalovirus, Adenovirus, Epstein Barr, Lyme, Streptococcal, Brucella, Mycoplasma, Legionella, Erchilicia, Tuberculosis, HIV, Cryptococcus, and COVID-19, were negative. The CT scan of the chest and abdomen with contrast was normal. A bone marrow aspirate and biopsy did not reveal any abnormal cells or hemophagocytes. The repeat blood tests showed rising inflammatory markers. Her hemoglobin and platelet counts dropped further (Figure 1).

**Figure 2:**
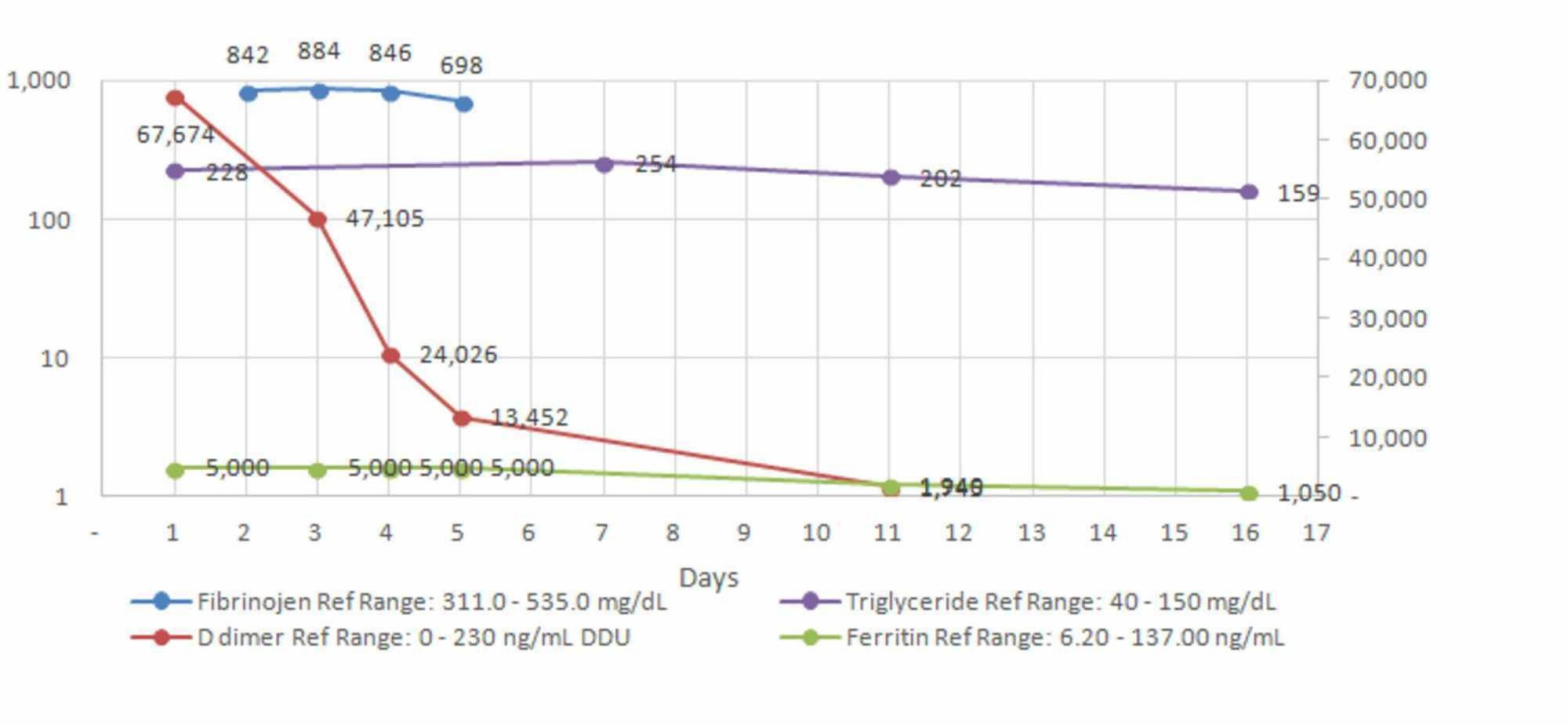
Trends of laboratory values of ferritin, d-dimer, fibrinogen, and triglyceride with time The left axis reflects mg/dl for fibrinogen and triglyceride and the right axis reflects ng/dl for ferritin and d-dimer.

There was a strong suspicion of COVID-19 with cytokine storm - multisystemic inflammatory disease in children (MIS-C) - because of the persistence of fever, pancytopenia, and transaminitis [2-3]. Secondary hemophagocytic lymphohistiocytosis (sHLH)/macrophage activation syndrome (MAS) due to collagen vascular diseases were also considered. We conducted further investigations to establish the diagnosis: Ferritin was markedly elevated to >5000 ng/ml (ref 6.2-137 ng/ml), D-dimer 67674 ng/dl (ref 0-230 ng/dl), triglyceride 254 mg/dl (ref 0-150 mg/dl), fibrinogen 842 mg/dl (ref 311-535mg/dl), and soluble IL 2 R alpha 4445 pg/ml (Figure 1B). Prothrombin time (PT)/activated partial thromboplastin time (APTT)/international normalized ratio (INR), troponin, atrial natriuretic peptide, electrocardiogram, and echocardiogram were normal. Urine protein/creatinine ratio was high with 24-hour urinary protein in the nephrotic range 3320 mg/24 hours. Trends of fibrinogen, d-dimer, ferritin, and triglyceride with time are seen in Figure 2. 

**Figure 3:**
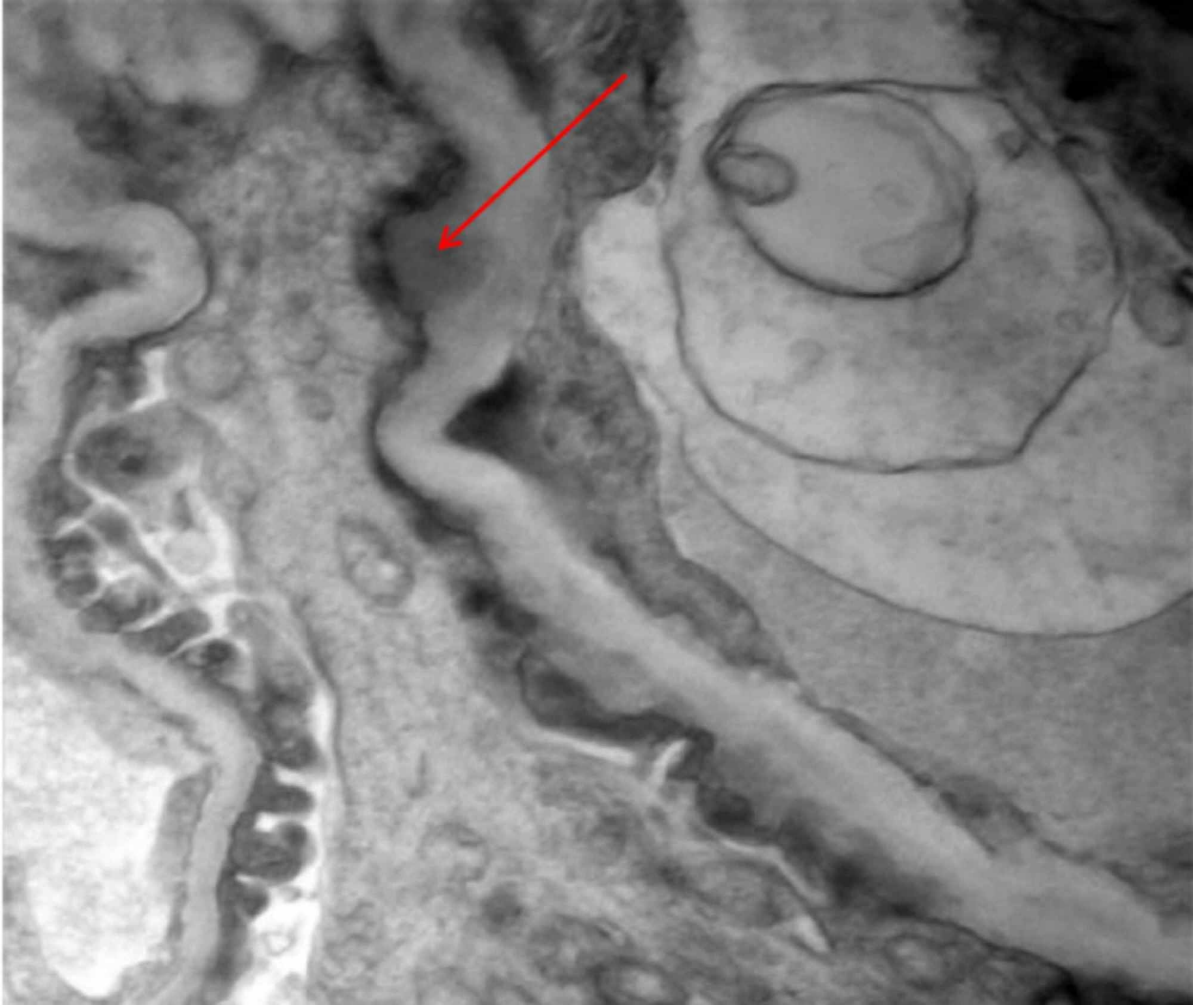
Electron microscopy of kidney biopsy showing subepithelial dense deposits

**Figure 4:**
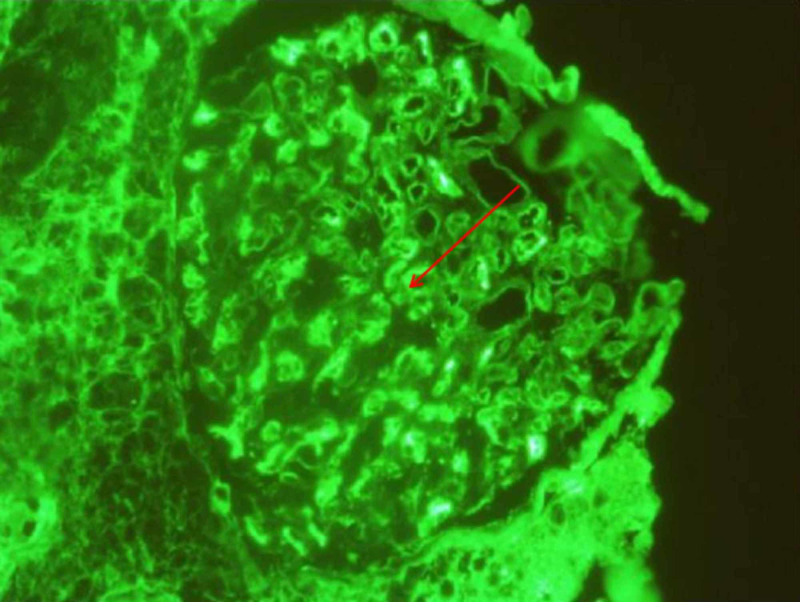
Immunofluorescence microscopy view of the kidney showing dense deposits

Our patient had five of eight hemophagocytic lymphohistiocytosis (HLH)-2004 criteria: fever>101.3°F, cytopenia, hyper-ferritinemia, hypertriglyceridemia, and elevated sIL2R. With the above laboratory results, sHLH was high on our list of possible diagnoses. COVID-19 with a cytokine storm (MIS-C) can present similarly with a significant overlap of manifestations [2-3]. Her antinuclear antibodies (ANA) were highly positive, 1:1260 (ref 1:60) with a nuclear speckled pattern, along with positive anti-smith and anti-sm/ribonucleoprotein (RNP) antibodies. Antiphospholipid antibodies, double-stranded deoxyribonucleic acid (dsDNA) anti-Sjögren's syndrome type A antigen A (anti-SS-A)/Sjögren syndrome type B antigen (SS-B), Perinuclear antineutrophil cytoplasmic antibodies (pANCA), cytoplasmic ANCA (cANCA), and anti-scleroderma 70 were negative. C3 and C4 complement levels were normal. An ultrasound-guided kidney biopsy showed moderate effacement of foot processes with infrequent subepithelial immune type electron-dense deposits consistent with membranous glomerulonephropathy (Figures 3-4). Phospholipase A2 receptor antibodies (PLA2R) were negative.

Based on the clinical and laboratory criteria, she was diagnosed to have systemic lupus erythematosus (SLE) with type V lupus nephritis (membranous nephropathy).

The patient was treated with pulsed intravenous solumedrol and enalapril. Her fever subsided within 24 hours after starting steroids, and she was discharged with follow-up appointments with a rheumatologist, nephrologist, and ophthalmologist.

## Discussion

SLE is a chronic inflammatory condition affecting multiple organs, and it affects females more than males. The clinical course of the disease is variable and patients may not present with all the signs at the same time. The clinical criteria are persistent fever, malar or discoid rash, photosensitivity, oral ulcers, serositis, psychosis, and chorea. Laboratory evidence includes hemolytic anemia, leukopenia, thrombocytopenia, false-positive venereal disease research laboratory (VDRL), renal involvement with proteinuria > 500 mg/24 hours (0.5 g/24 hrs), with positive serology (dsDNA, low C3 and C4, anti-Smith, anti-phospholipid antibodies). Proteinuria is the most common finding in lupus nephritis, which determines the long-term prognosis. Complications can result in end-stage renal disease with increased mortality.

HLH can be primary (autosomal recessive inheritance of gene mutation) or secondary to an infection, malignancy, and autoimmune diseases [4-5]. HLH secondary to rheumatological conditions or MAS has a prevalence of 0.9% to 4.5% [6]. The condition leads to loss of control of natural killer cells and cytotoxic lymphocytes over macrophages resulting in excessive immune activation and creating a cytokine storm destroying tissues [3-5]. It typically has a high d-dimer and a very high ferritin level, indicating the crucial role of macrophages in haem metabolism. Children who develop MAS secondary to a rheumatic disease can have high mortality rates from 5%-40% [6].

The 2019 European League Against Rheumatism (EULAR) and the American College of Rheumatology (ACR) emphasized that positive ANA must be an entry criterion for the diagnosis of SLE, plus clinical criteria to have a weighted score of >10 [7]. Our patient had a weighed score of 20 using the following: fever (score 2), thrombocytopenia (score 4), anti-Smith antibody (score 6), class V lupus nephritis (score 8). The dsDNA was negative with a normal complement level, but anti-Smith antibodies and anti-sm/RNP antibodies were positive.

## Conclusions

SLE is a multisystemic disease that is varied in its presentation and potentially a cause of pyrexia of unknown origin. There can be a significant clinical overlap between MAS secondary to SLE and COVID-19 infections with a cytokine storm (MIS-C). Patients with SLE nephritis may have normal complement levels and negative dsDNA. It is important to look for other antibodies like anti-sm/RNP antibodies and anti- smith antibodies. A renal biopsy will determine the final diagnosis.
